# Decentralized Privacy-Preserving Data Aggregation Scheme for Smart Grid Based on Blockchain

**DOI:** 10.3390/s20185282

**Published:** 2020-09-15

**Authors:** Hongbin Fan, Yining Liu, Zhixin Zeng

**Affiliations:** 1 College of Software and Communication Engineering, Xiangnan University, Chenzhou 423000, China; hongbinfan@xnu.edu.cn; 2 College of Computer Science and Technology, Hengyang Normal University, Hengyang 421002, China; 3 School of Computer Science and Information Security, Guilin University of Electronic Technology, Guilin 541004, China; bestzengzx@gmail.com

**Keywords:** decentralized, data aggregation, privacy-preservation, blockchain

## Abstract

As a next-generation power system, the smart grid can implement fine-grained smart metering data collection to optimize energy utilization. Smart meters face serious security challenges, such as a trusted third party or a trusted authority being attacked, which leads to the disclosure of user privacy. Blockchain provides a viable solution that can use its key technologies to solve this problem. Blockchain is a new type of decentralized protocol that does not require a trusted third party or a central authority. Therefore, this paper proposes a decentralized privacy-preserving data aggregation (DPPDA) scheme for smart grid based on blockchain. In this scheme, the leader election algorithm is used to select a smart meter in the residential area as a mining node to build a block. The node adopts Paillier cryptosystem algorithm to aggregate the user’s power consumption data. Boneh-Lynn-Shacham short signature and SHA-256 function are applied to ensure the confidentiality and integrity of user data, which is convenient for billing and power regulation. The scheme protects user privacy data while achieving decentralization, without relying on TTP or CA. Security analysis shows that our scheme meets the security and privacy requirements of smart grid data aggregation. The experimental results show that this scheme is more efficient than existing competing schemes in terms of computation and communication overhead.

## 1. Introduction

With the rapid development of society and economy, people’s demand for electric energy is increasing, which requires that the power supply be more secure and stable. However, the traditional power system cannot keep up with the pace of technological change, the system architecture remains unchanged, which leads to the decline of power system stability and frequent safety accidents. It brings a lot of inconvenience to people’s lives and causes huge economic losses to the government and enterprises. For example, in 2012, a large-scale blackout occurred in India, affecting 670 million people. Due to the low efficiency and security of the traditional power system, it cannot meet the development needs of human society. Therefore, smart grids emerge as the times require a new generation of power networks.

Smart grid is a fully-automated transmission network based on the physical grid system, which combines sensor measurement, computer, information communication, and automatic control technology [1]. The information flow between suppliers and users in smart grid is bidirectional, while the traditional power grid adopts the unidirectional centralized system. Users can control the intelligent use of household appliances and equipment at any time according to the floating situation of electricity price in different time periods. Suppliers can automatically monitor the grid, prevent power outages, optimize grid performance, etc. Although, compared with the traditional power grid, smart grid has many excellent characteristics. However, it is easy to cause the leakage of user electricity consumption data and identity information in the process of smart grid power data collection [2,3]. For example, the blackout notification software of Vector was attacked in 2018, resulting in the disclosure of private information of thousands of customers. With the continuous integration of network, information technology, and power system; network security has become an important part of energy and power security. For example, Ukraine’s power grid suffered the world’s first large-scale blackout due to hacker attacks at the end of 2015, and the leakage of private information brings great security risks to the power grid and users.

In order to deal with the leakage of power consumption data and identity privacy, data aggregation, secret sharing [4,5,6], differential privacy [7,8], and other schemes have been proposed by predecessors. Data aggregation is one of the most common methods to solve the security and privacy problems of smart grid. In [9,10,11,12,13,14,15,16,17,18,19], the scheme used encryption algorithms to aggregate the power consumption data of users, and hides the data of a single user in the data of other users to protect privacy. These schemes rely on a trusted third party or a central authority, but in fact the trusted third party or the central authority is not truly reliable, and the trusted third party or the central authority can be easily knocked down by malicious attackers and leak users’ private data.

Therefore, we propose a decentralized privacy-preserving data aggregation (DPPDA) scheme for smart grid based on blockchain, which is used to collect electricity consumption data without the trusted third party or the central authority in the smart grid.

We have summarized the contributions of our paper as follows:

(1) A decentralized data aggregation scheme based on blockchain is proposed. Blockchain is a new type of decentralized protocol that does not require a trusted third party or a central authority. Since the proposed scheme does not require the trusted third party or the central authority, this assumption will have a positive impact on reliability, and we can refrain from the malicious attack to the trusted third party or the central authority.
(2) The leader election algorithm is applied to select a smart meter from a residential area as a mining node (MN) to participate in the blockchain network. The MN uses Merkle hash tree to perform security authentication and data aggregation for smart meters in the residential area without any trusted third party.
(3) Paillier encryption, Boneh-Lynn-Shacham short signature, and SHA-256 function are applied to ensure the transparency of the blockchain data while achieving multiple privacy protections, which can effectively resist various security threats (such as replay attacks, tampering).

Note that the original idea has been presented in a conference [20]. In the current version, more detailed description is added to make it more easily understandable, for example, the design goals, MN election, and security analysis. Especially through the performance evaluation, it is proved that the proposed scheme is superior to the existing schemes.

The rest of this paper is organized as follows. In Section 2, the previous work in privacy-preserving data aggregation are introduced. In Section 3, blockchain, bilinear pairing, Boneh-Lynn-Shacham short signature, and the Paillier cryptosystem are given. In Section 4, the proposed system model is presented, and our scheme is proposed in Section 5. The security analysis is shown in Section 6. In Section 7, the performance of our scheme is evaluated. The research is concluded in Section 8.

## 2. Related Work

Privacy-preserving data aggregation in smart grids have attracted extensive attention of researchers. At present, the smart grid data aggregation solutions can be roughly divided into the following three categories.

The first category is data aggregation schemes based on traditional network architecture. Li et al. [9] proposed a privacy-preserving multi-subset data aggregation scheme (PPMA), their scheme based on Paillier cryptosystem, which enables the aggregation of electricity consumption data of different ranges. Liu et al. [10] proposed a privacy-preserving data aggregation without any TTP. This scheme uses EC-ElGamal to encrypt power consumption data and construct a virtual aggregation area for users with a certain degree of trust to shield the data of a single user. Guan et al. [11] proposed a flexible threshold for data aggregation based on the secret sharing scheme. This scheme adjusts the aggregation threshold according to the energy consumption information and time period of each specific residential area to ensure the privacy of personal data during the aggregation process, while supporting fault tolerance. Karampour et al. [12] proposed using Paillier encryption system and AV net mask to realize the aggregation of privacy protection data in smart grid can effectively protect the privacy of user data without any security channel. Chen et al. [13] proposed a data aggregation scheme based on Paillier encryption. The trusted authority generates a key for the meter to encrypt the consumed data of the meter. When a smart meter cannot work normally, the trusted authority provides the pseudo ciphertext related to the meter. The scheme solves the problem of meter failure to some extent, but it cannot completely solve the problem of privacy protection. In [14], a dynamic member data aggregation scheme based on identity signature and homomorphic encryption algorithm is proposed. The operation center obtains the sum of power consumption data in the virtual aggregation area, but knows nothing about the single user’s use data. This scheme reduces the complexity of a new user joining and old user exiting. However, the above research methods do not consider the trusted environment and used a trusted third party or central authority.

The second category is data aggregation mechanism based on fog computing architecture. Lu et al. [15] proposed a privacy protection data aggregation scheme based on fog computing. In this scheme, the fog device is used as the gateway between the internet-of-things device and the control center. Lyu et al. [16] proposed a privacy-preserving aggregation scheme with the aid of fog computing architecture. This solution uses differential privacy to count user data, thereby ensuring data confidentiality. Zhu et al. [17] proposed a privacy-preserving data aggregation scheme for fog-based smart grid. Blind signature and short randomizable signature are used to provide anonymous authentication, and then fog node is used to solve the billing problem after anonymous authentication. All user data in the above solutions are concentrated in the fog layer, which inevitably brings about the problem of centralization.

The emergence of blockchain technology provides a solution to the trusted third party and centralization problems because of its decentralized characteristics. Currently, there are several studies using blockchain as privacy-preserving method for data aggregation. Guan et al. [18] proposed a privacy-preserving data aggregation scheme for power grid communications. The study divided users into different groups and each group has a private blockchain. The study uses multiple pseudonyms to hide users’ identity. In this scheme, a key management center (KMC) is used to generate multiple public and private keys for users, which does not realize decentralization. Fan et al. [19] proposed a smart grid data aggregation based on consortium blockchain, and its signcryption algorithm can be applied to multidimensional data collection in the consortium blockchain. CC is the trusted third party of the scheme, which can realize key user monitoring and data recovery.

## 3. Preliminaries

In this section, we briefly introduce the necessary background.

### 3.1. Blockchain

Blockchain technology was first proposed in 2008 by Satoshi Nakamoto for Bitcoin [21]. Blockchain technology has been widely used in payment, internet of things, healthcare, finance, and so on [22]. Blockchain is a decentralized distributed ledger database maintained by network-wide nodes [23], which comprise of a chain of different data blocks in a chronological order. All hash data added to the block is immutable. Blockchain is a new application mode of consensus mechanism, distributed data storage, encryption algorithm, and so on. The miners are responsible for creating blocks, and each block in the blockchain is identified by a hash in the header. The hash is generated by the SHA-256 hash algorithm, which uses plaintext of any size and computes a 256-bit encrypted hash of fixed size. Each header contains the address of the previous block in the chain. The information in the block cannot be deleted or changed. Blockchain has the characteristics of decentralization, anonymity, security, reliability, non-forgery, tamper resistance, and so on. Its key technologies include block structure, Merkle tree, P2P network, hash function, timestamp, asymmetric encryption mechanism, etc. [24].

(1) Merkle tree. Merkle tree is a tree that stores hash values, also known as hash tree. The value of the Merkle tree leaf node is the hash value of the data block. The value of a non-leaf node is the hash of its corresponding child node concatenation string. Merkel root is the root value of the hash tree calculated by all transactions in the current block.
(2) SHA-256. SHA-256 is the most widely used cryptographic secure hash algorithm (SHA) in the blockchain, which is used to maintain the data integrity within the block. It provides a unique 256-bit hash code, also called data file signature.
(3) Timestamp. The blockchain uses timestamp to realize that all recorded transaction data are encoded by time information, which ensures the traceability and verifiability of the recorded data in the database. The “timestamp” technology makes the blockchain database non-tamperable and unforgeable, so it is also called proof-of-existence of the block data.

### 3.2. Bilinear Pairing

$G_{1}$ and $G_{2}$ are two q-order prime cyclic additive groups. $e:G_{1}\times G_{1}\to G_{2}$ is a bilinear mapping [25,26] that has the following properties.

(1) Bilinearity: $e(u^{a},v^{b})=e{\left(u,v\right)}^{ab}$ for all $u,v\in G_{1}$, and $a,b\in Z_{q}^{*}$.
(2) Non-degeneracy: for all $u,v\in G_{1}$, e(u,v)≠1.
(3) Computability: there exists an efficient algorithm to compute e(u,v) for all $u,v\in G_{1}$.

### 3.3. Boneh-Lynn-Shacham Short Signature

Boneh-Lynn-Shacham (BLS) short signature [27] scheme is a typical bilinear pairing scheme, which uses SHA-256 hash function $H_{1}:{\left\{0,1\right\}}^{*}\to G_{1}$ and g is a random generator of $G_{1}$, and a bilinear map $e:G_{1}\times G_{1}\to G_{2}$. The BLS signature scheme is divided into three phases: key generation, signature, and verification.

(1) Key generation. The secret key $x\in Z_{q}^{*}$, and compute the public key PK=x⋅g.
(2) Signature. The plaintext $m\in G_{1}$, compute the signature σ=x⋅H(m).
(3) Verification. If e(σ,g)=e(H(m),PK), then the signature is verified. Otherwise fails.

### 3.4. Paillier Cryptosystem

Paillier cryptosystem [28] is a probabilistic public-key cryptosystem that uses asymmetric encryption algorithm, which can effectively implement homomorphic properties. The encryption algorithm satisfies homomorphism of addition and multiplication, and can operate directly on the ciphertext without needing to know the corresponding plaintext. Therefore, it is widely used in many privacy protection applications. It includes three algorithms: key generation, encryption, and decryption.

(1) Key generation. Randomly select two large primes p and q, where |p|=|q|=|κ|. Then calculate λ=lcm(p−1,q−1). Defined a function $L(v)=\frac{v-1}{N}$, where N=pq. Choose a generator $g\in Z_{N^{2}}^{*}$, and calculate $\mu ={\left(L(g^{\lambda }\;\mathrm{mod}\;N^{2})\right)}^{-1}\;\mathrm{mod}\;N$. The public key is (N,g), and the corresponding private key is (λ,μ).
(2) Encryption. Given a message $m\in Z_{N}$, choose a random number $r\in Z_{N}^{*}$, gcd(r,N)=1. The ciphertext is calculated as $C=Enc(m)=g^{m}\cdot r^{N}\mathrm{mod}\;N^{2}$.
(3) Decryption. Given the ciphertext $C\in Z_{N}$, the corresponding message is decrypted with the private key (λ,μ) as $m=Dec(C)=L(C^{\lambda \;}\mathrm{mod}\;N^{2})\cdot \mu \;\mathrm{mod}\;N$.

## 4. System Model

### 4.1. Communication Model

**Figure 1:**
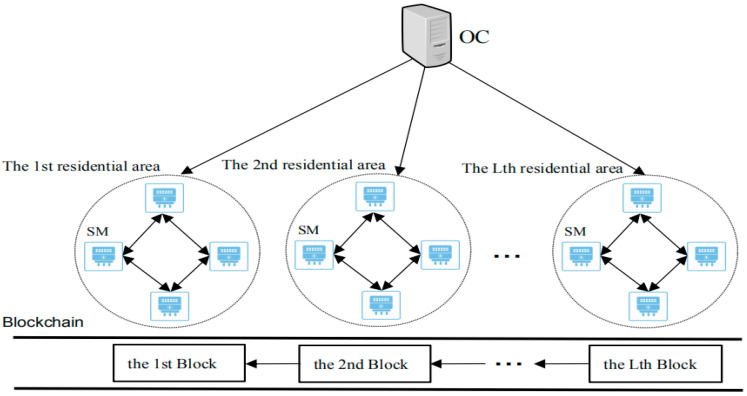
System model.

The system model of our scheme consists of operation center (OC) and smart meter (SM) in the residential area (RA), which is demonstrated in Figure 1. The system consists of L residential areas, and each residential area contains several smart meters. In our scheme, we mainly focus on removing the control center and the trusted third party while protecting the data privacy of the user’s smart meter.

(1) Operation center (OC). OC reads the real-time total power consumption data aggregated by the mining nodes of L blocks through the blockchain. OC can also perform billing, power consumption trend analysis, adjustment of power generation plans, and dynamic pricing. OC is vulnerable to attacks by external adversary. Therefore, OC is not assumed to be trusted.
(2) Smart meter (SM). A SM is an electricity meter for each user’s site in the residential area. The smart meter regularly and simultaneously (e.g., every 15 min) collects the power consumption data of each user’s household electrical equipment. Peer-to-peer (P2P) communication is used between all SMs in each residential area. Each residential area uses leader election algorithm to select a smart meter from the smart meters as the mining node (MN), then each residential area constructs a block through a MN. The MN selected by the MN selection algorithm can replace a trusted third party or a trusted authority, it is responsible for generating system parameters, authenticates the legitimacy of the data transmitted by the smart meter, and aggregates the encrypted data. Then, SM encrypts all kinds of collected data and uploads it to the MN after a short period of time. SM is assumed to be honest-but-curious, which executes the operations according the protocol without launching the active attack. However, it perhaps tries to analyze the received data to infer some valuable information.

### 4.2. Design Goals

To solve the issues mentioned above, ensure the integrity and privacy of users’ power consumption data while decentralizing or not relying on the trusted third parties, the design goals include five aspects.

(1) Privacy-preservation. Neither OC nor any other user has access to other user’s data in the residential area. An external adversary cannot obtain the user’s power consumption data, even if he knows the ciphertext. Even if the adversary and OC collude with each other, they can’t get the power consumption data of a single user’s smart meter.
(2) Decentralizing. Our scheme does not need a trusted third party or a central authority. The leader election algorithm is used to select a smart meter in the residential area as the mining node, which is responsible for building the Merkle tree of the block and aggregating the power consumption data of the residential area.
(3) Data unforgeability and non-repudiation. Our scheme adopts BLS short signature in blockchain, which is based on bilinear pair to ensure the unforgeability and non-repudiation of data.
(4) Data security. The proposed scheme can defend against various attacks. Even if the aggregate ciphertext of users’ electricity consumption data is intercepted, the individual user’s electricity consumption data cannot be recovered.
(5) Confidentiality. The data of electricity consumption belongs to personal privacy, which can reflect the real-time power consumption of users’ homes. Once the data is leaked, it will be used by criminals to commit crimes. Data confidentiality should be maintained by a secure data aggregation scheme. Even if an attacker steals the ciphertext, it will not be able to obtain the power consumption data of a single user.

## 5. The Proposed Scheme

In this section, a decentralized smart grid privacy protection data aggregation scheme based on block chain is proposed, which consists of five phases: system initialization, ciphertext generation, ciphertext aggregation, ciphertext decryption, and data reading. The notations are listed in Table 1.

**Figure 2:**
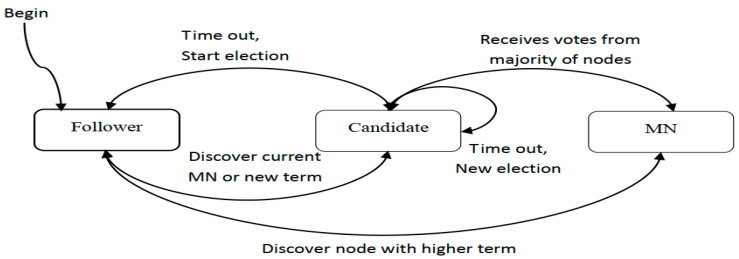
State transition model of MN election algorithm.

Each smart meter in the system acts as a node, and each node has three states: follower, MN, and candidate. All nodes start from the follower state. Each term begins with an election in which one or more candidates try to become MNs. If a candidate wins the election, it will be a MN for the rest of its term. The state change of MN election algorithm is shown in Figure 2.

### 5.1. System Initialization

**Figure 3:**
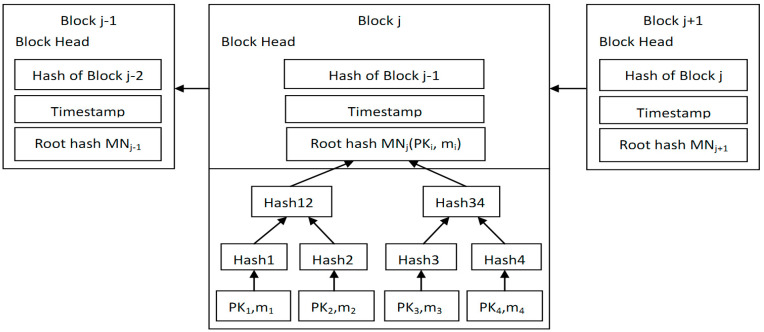
Blockchain structure in our scheme.

OC collects electricity consumption data of smart meters in L residential areas. There are n smart meters in $RA_{j}$. Through Algorithm 1, it selects a SM as a mining node from the *n* SMs in $RA_{j}$, then constructs the *j*th block, where $MN_{j}$ is the root of the Merkle tree in the *j*th block. The consumption data of SMs in $RA_{j}$ is aggregated to $MN_{j}$ through Merkle tree. The structure of blockchain is shown in Figure 3.
**Algorithm 1.** MN Election1.  Set the initial state of SM[*i*] to ***Follower***,*i*∈[1,*n*],*n* is the number of SMs in RA;2.  Let the number of terms of SM[*i*] elected as MN be 0,*TN* = 0;3.  Set the number of votes obtained by SM[*i*] to 0,*Nv* = 0;4.  Start the Timer of Follower *FT*;5.  Set a random timeout of Follower *FRT_out_*;6.  **while**
*FT > FRT_out_*
**do**7.  The state of SM[*i*] has changed from ***Follower*** to ***Candidate***;8.  *TN* = *TN* + 1;9.  Start the Timer of Candidate *CT*;10.  Set a random timeout of Candidate *CRT_out_*;11.  *Nv* = *Nv* + 1;12.  SM[*i*] with Candidate state sends a request of voting to other SMs;13.  SM[*i*] counts the number *k* of voting responses received from other SMs;14.  *Nv* = *Nv* + *k*;15.  **if**
*Nv* > *n*/2 + 1 **then**16.  The state of SM[*i*] has changed from *Candidate* to *MN*;17.  SM[*i*] sends messages that are selected as MN to other SMs;18.  **end if**19.  **if** SM[*i*] receives messages from a SM that is selected as MN **then**20.  The state of SM[*i*] has changed from *Candidate* to *Follower*;21.  **end if**22.  **while**
*CT > CRT_out_*
**do**23.  Repeat step 8–11 for a new election24.  **endwhile**25.  **endwhile**

$MN_{j}$ runs Bilinear parameter generator Gen(κ) to generate $\left(q,g_{1},G_{1},G_{2},e\right)$, and $g_{1}$ is a generator of $G_{1}$. $MN_{j}$ calculates Paillier cryptosystem public key $\left(N,g_{2}\right)$, corresponding private key (λ,μ), $g_{2}\in Z_{N^{2}}^{*}$. $MN_{j}$ choose a SHA-256 hash function $H_{1}$ and a secure cryptographic hash function $H_{2}$, where $H_{1}:{\left\{0,1\right\}}^{*}\to G_{1}$, $H_{2}:{\left\{0,1\right\}}^{*}\to {\left\{0,1\right\}}^{\kappa }$.

$MN_{j}$ publishes the system public parameter $\left\{q,g_{1},g_{2},G_{1},G_{2},e,N,H_{1}\right\}$.

### 5.2. Ciphertext Generation

Step 1 $SM_{i}$ selects a random number $x_{i}\in Z_{q}^{*}$ as the private key and computes the corresponding public key $PK_{i}=x_{i}\cdot g_{1}$.
Step 2 $SM_{i}$ collects electricity consumption data $m_{i}$ at timestamp *T*, and computes the Hash value $H_{2}\left(T\right)$, then selects a random number $r_{i}\in Z_{N}^{*}$ to generate ciphertext:
  $C_{i}=g_{2}^{m_{i}}\times {\left(r_{i}\times H_{2}(T)\right)}^{N}\mathrm{mod}\;N^{2}$.
Step 3 $SM_{i}$ generates the BLS short signature $\sigma_{i}=x_{i}\cdot H_{1}(C_{i}∥PK_{i}∥Ts_{i})$, $Ts_{i}$ is the current timestamp to prevent replay attack.
Step 4 $SM_{i}$ sends $C_{i}∥PK_{i}∥Ts_{i}∥\sigma_{i}$ to MN through the Merkle tree.

### 5.3. Ciphertext Aggregation

After $MN_{j}$ receives users’ data $C_{i}∥PK_{i}∥Ts_{i}∥\sigma_{i}$, it performs the following steps for privacy-preserving data aggregation.

Step 1 $MN_{j}$ verifies n signatures after receiving $C_{i}∥PK_{i}∥Ts_{i}∥\sigma_{i}$. If $e(\sigma_{i},g_{i})=e(H_{1}(C_{i}∥$$PK_{i}∥Ts_{i}),PK_{i})$ validation is successful and fails otherwise. If it holds, the signature is valid and $MN_{j}$ will accept $SM_{i}$’s ciphertext.
  In order to make the verification more efficient, $MN_{j}$ adopts batch verification
  $$e(\sigma_{i},g_{1})\underline{\underline{?}}e(H_{1}(C_{i}∥PK_{i}∥Ts_{i}),PK_{i})$$
  The proof is given as follows.
  $$\begin{array}{ll} e(\sum_{i=1}^{n}\sigma_{i},g_{1}) & =e(\sum_{i=1}^{n}x_{i}\cdot H_{1}(C_{i}∥PK_{i}∥Ts_{i}),g_{1})\\ & =\prod_{i=1}^{n}e(x_{i}\cdot H_{1}(C_{i}∥PK_{i}∥Ts_{i}),g_{1})\\ & =\prod_{i=1}^{n}e(H_{1}(C_{i}∥PK_{i}∥Ts_{i}),x_{i}\cdot g_{1}) \end{array}$$
Step 2 $MN_{j}$ aggregates the ciphertext.
  $$\begin{array}{ll} C=Enc(m) & =\prod_{i=1}^{n}C_{i}\;\\ & =\prod_{i=1}^{n}g_{2}\cdot (r_{i}\cdot H_{2}(T){)}^{N}{\;\mathrm{mod}\;N}^{2}\\ & =g_{2}^{\sum_{i=1}^{n}m_{i}}\cdot \prod_{i=1}^{n}(r_{i}\cdot H_{2}(T){)}^{N}{\;\mathrm{mod}\;N}^{2} \end{array}$$

### 5.4. Ciphertext Decryption

$MN_{j}$ uses the private key (λ,μ) to decrypt the aggregated ciphertext to obtain the aggregated electricity consumption data $M_{j}$ of the jth residential district.

$$\begin{array}{ll} M_{j} & =Dec(C)=L(C^{\lambda }{\;\mathrm{mod}\;N}^{2})\cdot \mu \;\mathrm{mod}\;N\;\\ & =\frac{L(C^{\lambda }{\;\mathrm{mod}\;N}^{2})}{L(g_{2}^{\lambda }{\;\mathrm{mod}\;N}^{2})}\;\mathrm{mod}\;N\;\\ & =\frac{L(g_{2}^{\sum_{i=1}^{n}m_{i}\lambda }{\mathrm{mod}\;N}^{2}{}^{\;})}{L(g_{2}^{\lambda }{\mathrm{mod}\;N}^{2}{}^{\;})}\mathrm{mod}\;N \end{array}$$

### 5.5. Data Reading

$MN_{j}$ generates the (j+1)th block, and adds the jth block to the blockchain after the (j−1)th block. OC obtains the power consumption data through the public key read blockchain.

## 6. Security Analysis

The security of DPPDA in smart grid is compared with that of schemes [9,11,12,13], as shown in Table 2.

### 6.1. Privacy-Preservation

To avoid the leakage of the power consumption data $m_{i}$, we mainly consider the external attack and the internal attack.

First, we assume that the external adversary may eavesdrops the communication between SMs and MN to obtain the electricity consumption data $m_{i}$. In DPPDA, $SM_{i}$ reports $m_{i}$ to $MN_{j}$ in the form of $C_{i}=g_{2}^{m_{i}}\times {\left(r_{i}\times H_{2}(T)\right)}^{N}\mathrm{mod}\;N^{2}$. Let $r=r_{i}\times H_{2}(T)$, then the ciphertext expression will become $C_{i}=g_{2}^{m_{i}}\times r^{N}\mathrm{mod}\;N^{2}$. The ciphertext $C_{i}$ is still the legal ciphertext of the Paillier cryptosystem. Because the adversary does not know the decryption key λ of the Paillier encryption algorithm, the adversary cannot decrypt the ciphertext $C_{i}$ to obtain the power consumption data of a single user. The power consumption data of a single smart meter is not disclosed, so as to protect the privacy of users.

Second, we assume that the internal adversary includes $SM_{1}$, $SM_{2}$, ···, $SM_{n-1}$, and they collude to obtain the power consumption $m_{n}$ of $SM_{n}$. The expression of *n* SMs is expressed as: $\sum_{i=1}^{n}m_{i}=0\;\mathrm{mod}\;\lambda$. For (*n*−1) users, the expression can be rewritten as: $m_{n}+\sum_{i=1}^{n-1}m_{i}=0\;\mathrm{mod}\;\lambda$. This means without having Paillier’s secret key λ, the internal adversary will not be able to obtain $m_{n}$. We can conclude that, no matter how many SMs are colluded, the internal adversary cannot disclose the power consumption data $m_{i}$ of the other users.

### 6.2. Decentralized

In our scheme, the blockchain can be implemented without a trusted third party or central authority, the availability and reliability of data is guaranteed by MN election. Any SM is not controlled or operated by other SMs and OC. P2P network is adopted among smart meters to realize decentralization. The whole process does not rely on a trusted third party to make our solution more reliable and convenient.

### 6.3. Data Security

The electricity consumption data of $SM_{i}$ in $RA_{j}$ is encrypted as $C_{i}=g_{2}^{m_{i}}\times {\left(r_{i}\times H_{2}(T)\right)}^{N}$ $\mathrm{mod}\;N^{2}$, *m_i_* is secure and privacy-preservation. Even if an adversary intercepts $C_{i}$, he/she cannot recover the power consumption data of a single smart meter. After MN collects all the smart meter power consumption data in the residential area through data aggregation, only the aggregated data can be obtained through decryption, and the plaintext of single smart meter power consumption data cannot be recovered.

### 6.4. Confidentiality

The power consumption data includes user privacy and business secrets. The usage data of the smart meters are encrypted by Paillier cryptosystem algorithm derived from [29]. After receiving the ciphertext of the smart meters in the residential area, only MN can decrypt the aggregated plaintext data. Since Theorem 1 of [28] represents confidentiality based on the DDH assumption, even if the adversary eavesdrops on the ciphertext of the smart meters in the residential area, the adversary still cannot infer any relevant information about usage data sent by the smart meters. The confidentiality of user power consumption data is guaranteed.

### 6.5. Data Integrity and Non-Repudiation

SHA is an anti-collision algorithm where different inputs (data information) cannot produce the same output (hash value), so SHA-256 can be used to check whether the data information is the same. The integrity of the data is determined by comparing the calculated “hash value” with the known hash value. Each smart meter in the scheme signs the message to be sent. MN receives the message after verifying the signature to ensure the integrity of the data and prevent tampering. Each smart meter’s private key is kept by itself and cannot be denied the information it sends and signs.

### 6.6. Data Unforgeability

All SMs use their private keys to sign their messages before sending MN use SM public keys to verify received messages. The proposed scheme uses the BLS signature based on the CDH [30], which makes it impossible for any attacker to forge a new signature by eavesdropping on the original signature. BLS short signature and blockchain are used to verify the source and authenticity of power consumption data. Since all transactions in the blockchain have timestamps and all hash data added to the block cannot be changed, the data in the blockchain has unforgeability.

## 7. Performance Evaluation

The performance of our scheme is evaluated in this section, including the computation complexity of SM and OC, and the communication overhead.

### 7.1. Computation Complexity

Compared with multiplication operation and exponentiation operation, leader election and hash operation is negligible. In our scheme, the computations in the data aggregation process mainly include three phases, data encryption, batch verification, and aggregation, decryption. We denote the computational cost of an exponentiation operation and a multiplication operation, by $T_{exp}$, $T_{mul}$, respectively. The computation complexities of the major entities in the system are as show in Table 3.

**Figure 4:**
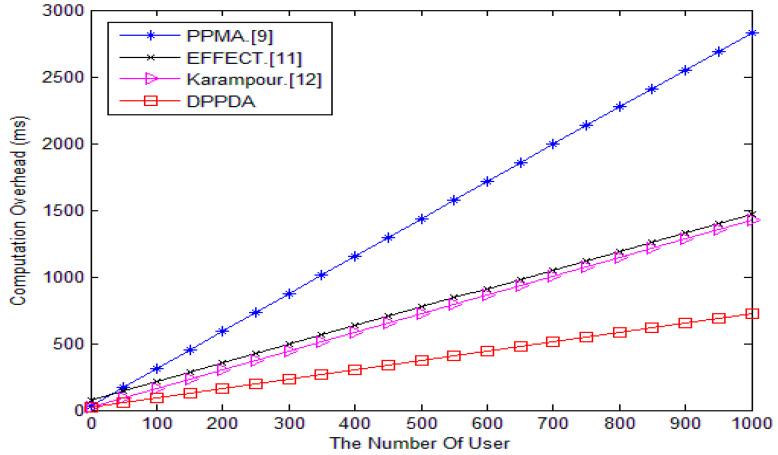
Comparison of computational cost.

We conduct the experiments with the cpabe0.10 [31] library on a 3.0-GHz processor and a 2-GB memory PC. As shown in Figure 4, compared with PPMA, EFFECT, and Karampour’s schemes, our scheme has much less computational overhead. As the number of users increases, the advantages of our scheme become more obvious.

### 7.2. Communication Overhead

The communication of our proposed scheme is only $SM_{i}$ to $MN_{j}$, the power consumption data collected by $SM_{i}$ is used to generate the report $\left(C_{i}∥PK_{i}∥Ts_{i}∥\sigma_{i}\right)$ and is sent to $MN_{j}$. The size of $SM_{i}$ report is $S_{z}=|C_{i}|+|PK_{i}|+|Ts_{i}|+|\sigma_{i}|$. The maximum communication overhead is $S_{\max}=$$n\cdot S_{z}=n\cdot (|C_{i}|+|PK_{i}|+|Ts_{i}|+|\sigma_{i}|)$. Suppose that

$$SM_{i}$$

generates a 2048-bit ciphertext

$$C_{i}$$

and chooses 160-bit $Z_{N}^{*}$, 160-bit G.

In PPMA and EFFECT scheme, the communication cost on SM-to-GW is 2048*n* bit, the communication cost on GW-to-CC is 2048 bit, the total communication overhead is 2048(*n* + 1) bit. In Karampour’s scheme, the communication cost on SM-to-SM is *n*(2048(*n* − 1)) bit, the communication cost on SM-to-GW is 2048*n* bit, the communication cost on GW-to-CC is 2048 bit, the total communication overhead is 2048(*n*^2^ + 1) bit.

In our scheme, the total communication overhead is 2048*n* bit. The comparison is shown in Table 4, the total communication cost of our scheme is less than the other schemes.

**Figure 5:**
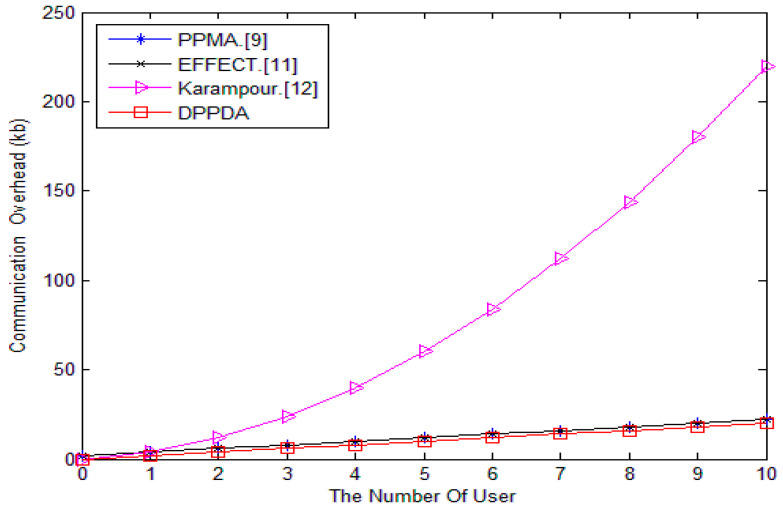
Comparison of communication cost.

In Figure 5, we plot the communication cost in PPMA, EFFECT, Karampour’s, and our scheme versus the SM number *n*. It is shown that our scheme does not bring too much communication overhead.

## 8. Conclusions

In this paper, a decentralized smart grid privacy-preservation data aggregation scheme based on blockchain is proposed. The smart meters select a mining node through leader election algorithm, which records the data of smart meters into the blockchain. BLS signature and Paillier encryption are based on bilinear pairing, which guarantees the security and integrity of messages during transmission. Security analysis shows that our mechanism meets the requirements of privacy protection and security of smart meters. The performance evaluation shows that our scheme is superior to some popular data aggregation schemes in computational efficiency. Our scheme has low communication overhead and does not require any trusted third party, trusted authority, and secure channels. At present, we have decentralized the aggregation of one-dimensional power consumption data. In the future, we will work on the combination of blockchain and other algorithms to aggregate multidimensional power consumption data.

## Figures and Tables

**Table 1:** Notations.

Symbol	Quantity
$g_{1},g_{2}$	A generator of *G*
$RA_{j}$	the *j*th residential area
$m_{i}$	Power consumption data of the *i*th smart meter in $RA_{j}$
n	Number of smart meters in the *j*th residential area
$H_{1}$	Hash functions: $H_{1}$: {0,1}*→*G*
L	Number of residential areas
$SM_{i}$	Smart meter in *j*th residential area
$MN_{j}$	Mining node of the *j*th residential area
$M_{j}$	the aggregated electricity consumption data of the *j*th residential areas
‖	Concatenation operation

**Table 2:** Comparison between the proposed scheme and other related schemes.

Security Requirements	[9]	[11]	[12]	[13]	DPPDA
Blockchain-based	No	No	No	Yes	Yes
Decentralization	No	No	No	No	Yes
Non-repudiation	No	Yes	No	Yes	Yes
Privacy	Yes	Yes	Yes	Yes	Yes
Confidentiality	Yes	Yes	Yes	Yes	Yes
Data integrity	Yes	Yes	Yes	Yes	Yes
Replay attack resistance	No	Yes	Yes	Yes	Yes
Data unforgeability	No	Yes	Yes	Yes	Yes

**Table 3:** Comparing computation complexity between the proposed scheme and other schemes.

Scheme Ref.	[9]	[11]	[12]	DPPDA
Overhead SM	$3T_{exp}+4T_{mul}$	$4T_{exp}+3T_{mul}$	$2T_{exp}+nT_{mul}$	$2T_{exp}+4T_{mul}$
Overhead GW	$nT_{mul}$	$3T_{exp}+(2n+1)T_{mul}$	$nT_{mul}$	-
Overhead CC	$T_{exp}+(4n+3)T_{mul}$	$3T_{exp}+2T_{mul}$	$T_{exp}+3T_{mul}$	-
Overhead MN	-	-	-	$T_{exp}+(n+1)T_{mul}$

**Table 4:** Comparing communication cost between the proposed scheme and other schemes.

Scheme Ref.	[9]	[11]	[12]	DPPDA
SM-to-SM (bit)	-	-	*n*(2048 (*n* − 1))	-
SM-to-GW (bit)	2048*n*	2048*n*	2048*n*	-
GW-to-CC (bit)	2048	2048	2048	-
SM-to-MN (bit)	-	-	-	2048*n*
